# The Buffalo State Asylum for the Insane—Annual Report

**Published:** 1882-10

**Authors:** 


### THE BUFFALO STATE ASYLUM FOR THE INSANE—ANNUAL REPORT.

Through the courtesy of the Superintendent, we have been favored with a copy of the Eleventh Annual Report of this excellent institution, containing the annual statements of the Board of Managers, the Treasurer's report, and the report of the Superintendent. The pamphlet is full of valuable information, especially interesting to the profession, inasmuch as it presents a record of work in an important department of medicine, from the pen of Dr. Andrews, whom we regard as one of the ablest alienists of the country.

Referring to the results of treatment, the Superintendent makes these forcible remarks: "While in a majority of instances in which the disease has passed into a chronic state recovery cannot be looked for, still, much good can be derived from care and treatment in an asylum. The regularity of life and the favorable hygienic conditions under which patients are placed result in benefit to their general health and promote self-control, with such an amelioration of their condition that quite a proportion can return to their homes and enjoy the comforts and satisfaction of domestic life. * * * In view of the fact established by experience that the curability of insanity is in proportion to the *recent character* of the disease, the law makes it obligatory upon public officials to send cases which come under their jurisdiction, within ten days, to some asylum for treatment. This would seem to insure early care and treatment to all the insane of the public class before such changes occur in the brain as place the subject of them beyond the reach of medical skill and the curative measures adopted for their recovery. It may be asked why, under such circumstances, so many reach the chronic stage without treatment, and why, every year, the various asylums report so many chronic cases admitted? The answer is at hand. One potent cause is found in the fact that insanity is often slow and insidious in its inception and progress, and that its changes in character resemble, so closely, mere eccentricities of conduct that two or more years are passed before it is fully appreciated, and then, perhaps, only when the person becomes troublesome or violent. *Ignorance of what insanity is*, is a bar to its ready recognition."

This extract contains the experience of an accomplished expert, who has had exceptional opportunities to study insanity in all its phases, and is suggestive to the profession of the necessity of promptness, not only in the diagnosis of mental diseases, but in the removal of such patients to asylums, which are, in fact, hospitals for the treatment of mental diseases, under the personal supervision of physicians who make the study and treatment of this special class of diseases their life-work.

The report is quite full in tabulated statements from the records of the year, of the cases under treatment, which afford valuable data in regard to the forms of insanity admitted, the assigned causes, civil or social condition, degree of education, hereditary transmission, duration of insanity prior to admission, ages of the 219 cases admitted, supposed duration of insanity before admission, suicidal or homicidal tendencies, number of attacks in those admitted, nativity, occupation, causes of paresis in the nine cases admitted, etc. The tabulated statement of the hereditary transmission of insanity contains a suggestion which is worthy of consideration. Of the cases from which the family history could be traced, 38 per cent. traced their insanity to the maternal branch and 24 per cent. to the paternal branch. This shows how potent an influence the mother exerts in moulding the mental peculiarities of her offspring, a fact more generally accepted by the profession in regard to the physical conformation than the mental and intellectual.

We have not space to draw farther from this valuable report. It bears evidence of earnest and careful labor in this important department of medical science, and also demonstrates what we have before assured our readers, that our State Asylum possesses advantages, not only in the superiority of the arrangement and plan of its buildings, which admits of a thorough classification of patients, but also in the skill and attainments of its medical staff, whose efforts to raise the institution of whose interests, administrative and professional, they are the custodians, to the front rank in the special field assigned it, have met with the success which earnest and conscientious labor in a noble cause justly deserves.

The Superintendent pays a graceful compliment to his attendants and employees for their fidelity to duty and for their success in the management of the patients under treatment. We join in giving the endorsement of the profession to the excellent work which this report presents. Not only Dr. Andrews, but his accomplished assistants, Drs. Granger and Crego, by their ability and zeal, in the brief period during which the asylum has been open for its work, have established for the institution an enviable reputation in the successful management and treatment of the insane.

